# A comparative analysis of **GEANT4** , **MCNP6** and **FLUKA** on proton‐induced gamma‐ray simulation

**DOI:** 10.1002/mp.17754

**Published:** 2025-03-11

**Authors:** Hugo Freitas, Esmaeil Nobakht, Florian Grüner, Joao Seco

**Affiliations:** ^1^ Division of Biomedical Physics in Radiation Oncology German Cancer Research Center (DKFZ) Heidelberg Germany; ^2^ Department of Physics and Astronomy Heidelberg University Heidelberg Germany; ^3^ Department of Physics Hamburg University Hamburg Germany; ^4^ Institute for Experimental Physics and Center for Free‐Electron Laser Science (CFEL) University of Hamburg Hamburg Germany

**Keywords:** GEANT4, MCNP6, FLUKA, Monte Carlo simulations, prompt gamma‐ray spectroscopy

## Abstract

**Background:**

Precise range verification is essential in proton therapy to minimize treatment margins due to the steep dose fall‐off of proton beams. The emission of secondary radiation from nuclear reactions between incident particles and tissues stands out as a promising method for range verification. Two prominent techniques are PET and Prompt Gamma‐Ray Spectroscopy (PGS). PGS holds significant promise due to its real‐time capability for range monitoring. This method allows for prompt detection and quantification of any disparities between planned and actual dose delivery, facilitating adaptive treatment strategies. Given the key role of Monte Carlo (MC) codes in understanding the PGS mechanisms during proton therapy, it is essential to address the current lack of validated codes covering the full energy spectrum of emitted gamma‐rays.

**Purpose:**

Addressing the need for precise range monitoring in proton therapy, our study aims to develop and validate MC codes for PGS. We focus on analyse MCNP6, GEANT4, and FLUKA codes, conducting rigorous validation process by comparing our simulation results with experimental data. Additionally, we propose optimal models and parameters to refine the accuracy of simulations for prompt gamma‐ray (PG) spectra.

**Methods:**

Various proton data libraries, models and cross‐sections values were used in this study to simulate proton‐induced gamma‐rays in MCNP6, GEANT4 and FLUKA. To validate these simulations, PGS spectra of $15.0\;{\mathrm{cm}}^{3}$ PMMA block irradiation were obtained with ${\mathrm{CeBr}}_{3}$ inorganic scintillator detector for different proton energies, raging from approximately 90 to 130MeV.

**Results:**

GEANT4 was the only MC code capable of successfully reproducing 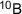 PG lines, while the FLUKA aligned better with experimental data for mid‐range energies. At higher energies, FLUKA overestimated the 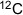 PG line ($2^{+}\to 0^{+}$) at 4.44MeV, whereas GEANT4 underestimated it; MCNP6 provided the closest match. Additionally, GEANT4, FLUKA, and MCNP6 failed to accurately reproduce the 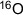 PG line ($3^{-}\to 0^{+}$) at 6.13MeV, consistent with previous findings. To address this limitation, a new model based on experimental and theoretical data from literature was developed.

**Conclusions:**

This study emphasizes the need for updates to the data tables in MC simulations and underscores the importance of further theoretical and experimental research on PG de‐excitation lines relevant to proton therapy. The newly developed model, designed to address discrepancies in the simulation of 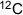 and 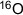 de‐excitation lines across different toolkits, successfully improved the accuracy of the oxygen de‐excitation line, which was previously not well‐reproduced.

## 1 INTRODUCTION

It is widely recognized that compared to photons, proton beams offer superior physical selectivity. This advantage is due to their unique dose deposition, which reaches its maximum (the *Bragg* peak) at an energy‐dependent depth before abruptly falling off. This characteristics allows for reduced entrance and exit doses compared to photon therapy.^1^ However, range uncertainties suffers from ambiguous conversion of *Hounsfield* units to stopping powers, limited accuracies in the pencil beam algorithm, patient motion, setup errors, and physiological changes over several weeks of fractionated therapy compromise the physical advantage of proton therapy.^2^ These uncertainties necessitate substantial safety margins to avoid large associated risks due to the steep *Bragg* peak dose gradient.^1^ Therefore, reduction of range uncertainties is crucial for maximizing the benefits of particle therapy, which can be achieved through the implementation of in vivo range monitoring techniques.

Prompt Gamma‐Ray Spectroscopy (PGS) has been proposed as an alternative to Positron Emission Tomography and is based on the detection of the secondary gamma‐ray radiation resulting from nonelastic nuclear interactions that escape the body. These gamma‐rays consist of prompt photons, which are emitted during the nuclear reactions, and delayed emission from the decay of unstable nuclear reaction products.^3^ However, the detection and collimation of these high‐energy gamma‐rays that are up to 10 MeV is still a challenging process. Given that each energy line in PGS can convey specific information, significant research and development efforts are needed to improve this method, in which simulation studies and Monte Carlo (MC) simulations in particular play an important role in advancing the understanding and effectiveness of PGS.

Reflecting the crucial role of MC simulations in the progress of range verification techniques, our study focused on developing models for GEANT4 v11.1.2,^4^, ^5^, ^6^ FLUKA ^7^, ^8^ and MCNP6 v2.^9^ We conducted a comparative analysis of these models with experimental results obtain at the Heidelberg Ion‐Beam Therapy (HIT) Center,^10^ aiming to improve the accuracy and effectiveness of PGS in clinical settings.

## 2 MATERIALS AND METHODS

**FIGURE 1:**
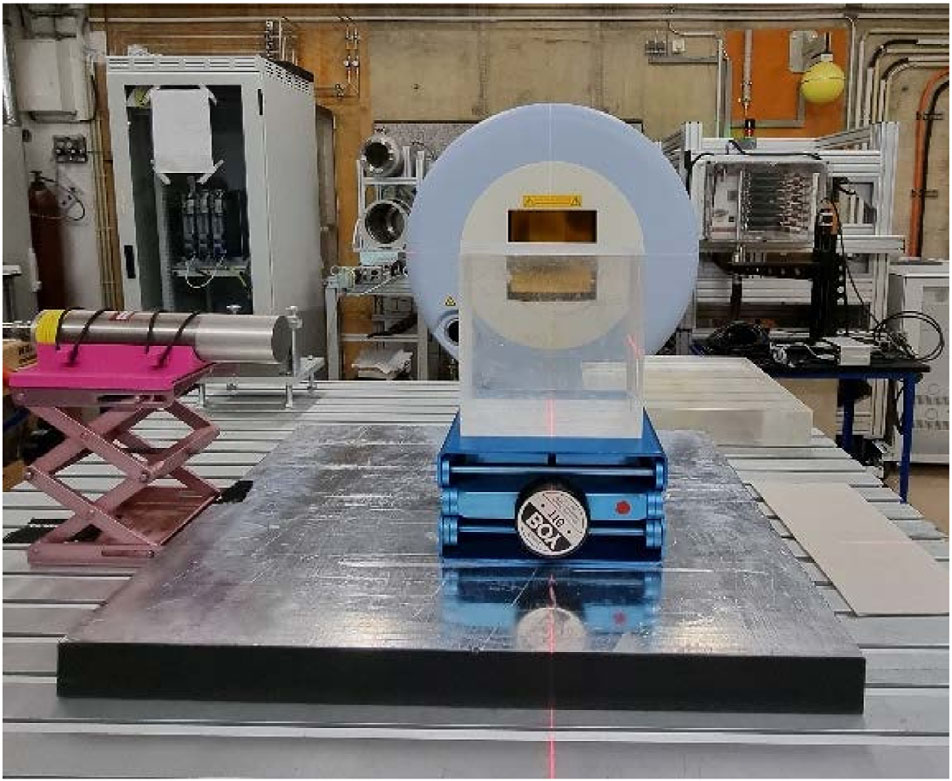
Experimental set‐up. The detector is on the left, the PMMA is in the middle, and the exit point of the proton beam is in the background. PAMM, polymethyl‐methacrylate.

The PG emission spectra of a PMMA target were experimentally investigated upon irradiation with a proton beam at the synchrotron‐based facility HIT. Figure 1 shows the general experimental setup used for the experimental measurements. The following sections provide a detailed description of the main components used in this study.

### 2.1 Materials

*PMMA blocks*. Polymethyl‐methacrylate (or PMMA) was used to assess changes in the spectra as a function of proton energy. In total, three blocks of size $15\times 15\times 5\;{\mathrm{cm}}^{3}$ were used. We aligned the blocks to obtain a tissue equivalent block thickness of 15cm. It should be also mentioned that the PMMA material was defined based on the study that has been done by Hünemohr et al.^11^

### 2.2 Detector system

*Detector*. A cylindrical‐shaped ${\mathrm{CeBr}}_{3}$ scintillating crystal with dimensions of 3.8cm in diameter and 7.62cm in length. This hygroscopy detector was coupled to a Hamamatsu photomultiplier (PMT) R9420‐100 and protected by layers of Teflon and aluminium.^12^

*Electronics*. The PMTs readout was performed with a high‐performance ADC, FlashCam FADC. The FlashCam FADC system has a multi‐channel acquisition mode capable of digitalizing data at 250 MS/s with 12‐bit resolution. It has independent channels that perform a readout of all channels as soon as a trigger is activated. All acquisitions in this study were performed event‐by‐event with single traces of Δt=200ns and sampling intervals of δt=4ns.

### 2.3 Experimental data analysis

From the interaction of the PGs with the primary detector, a current pulse proportional to the energy deposited arises. Each event time window in the ADC is 200ns and may measure one or more of these pulses. The PG spectrum is then obtained by a process similar to that used by Freitas et al.^13^ First, a hardware baseline restoration is applied, then an exponential modified Gaussian (EMG) is fitted to a maximum of 3 peaks. A spectrum can be built using either the areas or apex of the EMGs fits. Such spectrum is then corrected for dead time based on a non‐paralyzable model.^13^

To improve the quality of the spectrum, the spill structure was used to select the in‐spill events.^14^ Finally, in this study, a filter based on the adjusted *R‐squared* and the relaxation time of the EMGs was further applied to the spectrum. Such a filter was maintained between the same experimental sets and allowed the enhancement of the spectra peaks. For spectra calibration purposes, we followed a similar approach reported in other studies.^12^, ^15^, ^16^ A $137^{\mathrm{Cs}}$ radiation source (0.662MeV) was used along with the most prominent lines of PGS, including, annihilation peak (0.511MeV), hydrogen neutron capture peak (2.22MeV) and the PG line from the 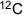 de‐excitation (4.44MeV) as well as its corresponding single and double escapes peaks were considered.

### 2.4 Monte Carlo simulations

Different MC codes were employed in our study to replicate experimental data. Following the initial simulations and data collection, a histogram representing the energy distribution of deposited photons was generated for each MC code. To model the detector's response function and energy resolution, a convolution function to the histogram was applied.^15^ This approach enabled a more precise representation of the detector's performance. Finally, to normalize our results and enable comparisons, the final PG spectra were divided by the total number of simulated particles and by the bin size in MeV. This normalization facilitated the interpretation and comparison of our data with experimental results.

#### MCNP6

Initially, only the proton data libraries were used. However, the gamma‐ray emission spectra in these datasets are very coarse and lack the precision required to identify specific gamma‐ray lines, especially in the low energy range. Therefore, all proton‐induced reactions were simulated using the physics models. It is generally assumed that when a proton strikes an atomic nucleus, it initiates a sequence of nuclear reactions including an intranuclear cascade (INC), pre‐equilibrium, evaporation or fission, and finally the de‐excitation of the residual nuclei through gamma‐ray emission. This process can be divided into two main stages, and there are several different models for modeling each stage: INC models predict nucleon‐nucleon collisions resulting in the emission of prompt particles. Subsequently, evaporation/fission models address some or all of the following: pre‐equilibrium, evaporation, fission, and the de‐excitation of residual nuclei.^17^ In MCNP6, the default INC model is *Bertini* for nucleons and pions, while the ISABEL model is used for other particle types.^18^ For this study, however, the ISABEL model was used for all incident particles type. Upon completion of the INC stage, the energy of the highly excited nucleus is dissipated by evaporation of neutrons, protons and light ions. This was achieved in our study by using the *Dresden* evaporation model combined with the Rutherford Appleton Laboratory fission model. Several discrete and continuous energy neutron data libraries were tested for neutron induced interactions. Regarding scattering gamma‐ray emissions, the default ACE model was changed to the *Cascading Gamma‐Ray Multiplicity* model, which has been integrated into the version 6.2 of MCNP6 code and has several advantages over the use of ACE data libraries.^19^ Finally, the pulse height tally *F8* was used, which records the number of pulses detected in user‐specified energy bin, analogous to a detector response function.

#### GEANT4

Our investigation begins by identifying the combinations of models and cross‐sections that are most suitable for this analysis. Among those recommended by GEANT4 for medical physics applications, we selected the following physics: *QBBC*, *QGSP_BIC_HP* and *QGSP_BIC_AllHP*. These physics lists were combined into a modular physics list with the *G4EmStandardPhysics_option4*. All physics lists are hybrid models that combine the *Bertini* pre‐compound models (for energies <6GeV) with the Binary Ion Cascade. The main differences lie in the cross sections values used and in the application of high precision models for neutrons (HP) and for both, neutrons and protons (AllHP).^4^, ^5^, ^6^ In our simulation settings, a custom scoring system capable of recording essential information about each interaction event was developed. This scoring system specifically recorded data on the amount of energy deposited in the detector per event, the time at which each event initially interacted with the detector, and the type of particle involved. To construct the PG spectrum, only the energy deposited by photons was exclusively considered, while neutrons and electrons were excluded from the analysis. Furthermore, the scoring process was also restricted to the active part of the detector to focus on the relevant interactions.

#### FLUKA

In the FLUKA toolkit, for the energy ranges employed in this study, the PEANUT package was used, including its General Intra‐Nuclear Cascade (GINC). This package provides a higher level of detail and consists of a comprehensive pre‐equilibrium stage followed by different equilibrium processes (e.g., evaporation, gamma de‐excitation). Inelastic cross sections for hadron‐hadron and hadron‐nucleus interactions are derived from experimental data and established data tables.^7^, ^8^ Unlike previous toolkits, FLUKA adopts certain default settings predefined for specific problems. For this study, we used two settings: *HADROTHE*, adapted for hadrotherapy calculations, and *PRECISION*, designed for precise simulations. The *DETECTOR* card was employed to gather all the PG spectra.

### 2.5 Experimental campaign

The experimental measurements carried out at HIT were used to benchmark and optimize the MC models. Figure 1 shows the measurements obtained by shooting a proton beam at the PMMA target, which was placed 100.0±0.5cm away from the nozzle. The PG detector was located 25.0±0.5cm away from the proton beam isocenter and perpendicular to the beam. We conducted a total of 5 proton irradiations with similar durations (∼150s) but different beam characteristics. Table 1 contains a summary of the beam information. The range of particles was determined using the calculated continuous slowing down approximation (CSDA) ranges at different energy values, with the assistance of NIST data.^20^

**TABLE 1:** Run number, initial proton kinetic energy in PMMA and beam FWHM and the corresponding CSDA range.

Run	Energy (MeV)	FWHM (cm)	RCSDA (cm)
1	89.91	1.75	5.514
2	99.74	1.58	6.633
3	110.24	1.44	7.923
4	120.05	1.34	9.212
5	129.52	1.25	10.53

Abbreviations: CSDA, continuous slowing down approximation; FWHM, full width at half maximum; PAMM, polymethyl‐methacrylate.

## 3 RESULTS

### 3.1 Experimental PG spectra

**FIGURE 2:**
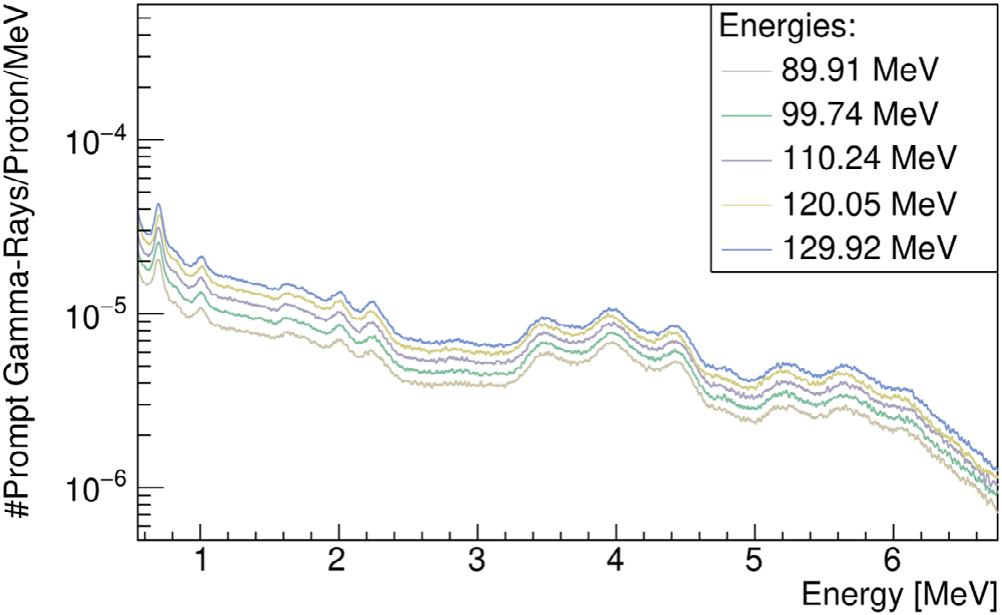
Prompt gamma‐ray spectra resulting from proton irradiation of PMMA. Measurements were obtained for different proton energies. PAMM, polymethyl‐methacrylate.

We started by irradiating the PMMA target with single‐spot and monoenergetic proton beams in the first set of experiments. Figure 2 shows the spectra obtained for the various beam energies used in this work. The most statistically relevant lines in the spectra, resulting from inelastic interactions of the proton with the target, include the boron (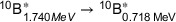, 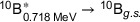), carbon (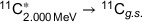, 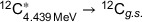), nitrogen (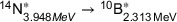, 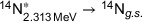), and oxygen de‐excitation lines (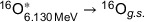).^21^ Additionally, we also have neutron activation of hydrogen (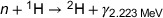).^15^

### 3.2 Monte Carlo PG spectra

**FIGURE 3:**
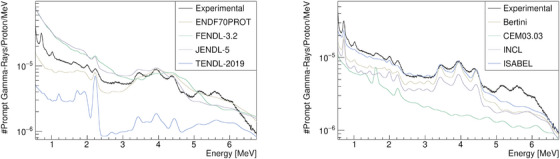
Simulated prompt gamma‐ray spectra in MCNP6 using various nuclear data tables (left) different intra‐nuclear cascade models (right).

**FIGURE 4:**
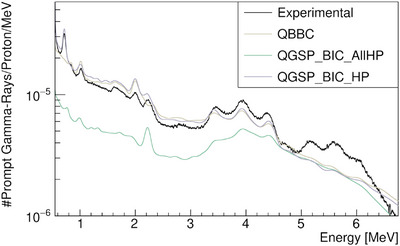
Simulated prompt gamma‐ray spectra in GEANT4 for various physics lists.

**FIGURE 5:**
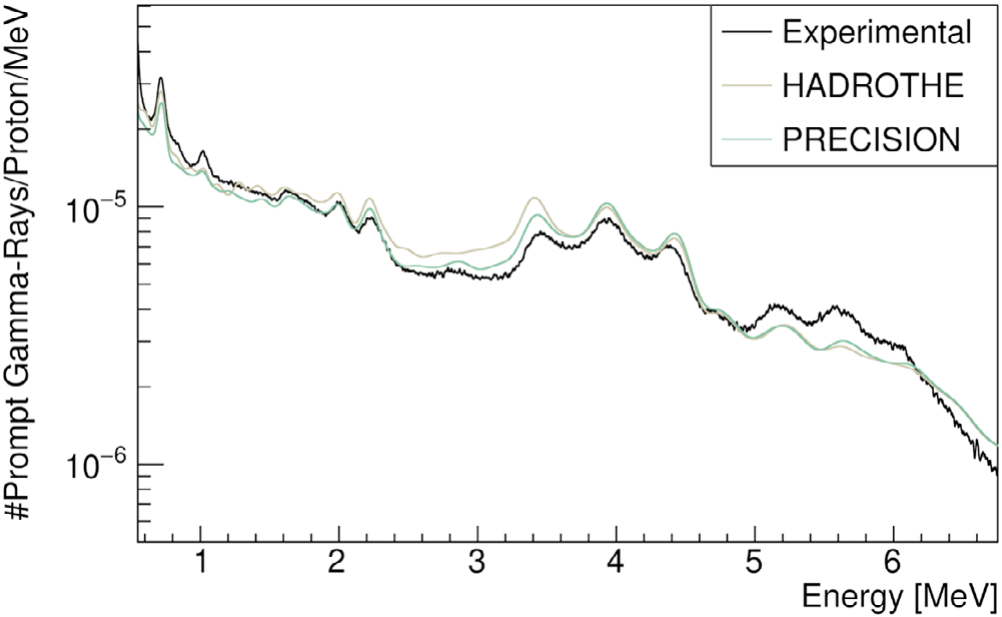
Simulated prompt gamma‐ray spectra in FLUKA for different defaults.

Our benchmark analysis began by selecting the most suitable options for MCNP6. We then ran the simulations using the data tables and models. The MCNP6 results, for the beam energy of $E_{beam}=110.24\;\mathrm{MeV}$, are shown in Figure 3. It is evident from the figure that data tables are inconsistent when compared to the experimental spectra. Therefore all the proton induced reactions were simulated using physics models. Among the various models tested, the ISABEL model provided the best fit to the data. However, none of the models could account for the oxygen de‐excitation line (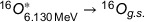) observed in the experimental results. In GEANT4 we have tested two models, the QBBC and the *QGSP_BIC* and its extensions. The results for both models and their extensions are depicted in Figure 4. The *QGSP_BIC_HP*, excluding background considerations, shows good agreement with the experimental data for energies below 3MeV. However, an exception is the 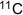 de‐excitation line, which is overestimated. Regarding the 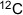 de‐excitation line, it is underestimated, which is consistence with findings in previous studies.^22^ Similar to the results from MCNP6, the oxygen de‐excitation line is also missing from the spectra. The results, depicted in Figure 5, exhibit similar behaviour between the two, with the former displaying a small artifact near the double escape peak of the carbon de‐excitation line (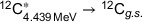). Although the oxygen de‐excitation line is more prominent in FLUKA, it is still significantly underestimated. Additional energies are provided in Figure S1.

### 3.3 EMPIRICAL Monte Carlo PG spectra

**FIGURE 6:**
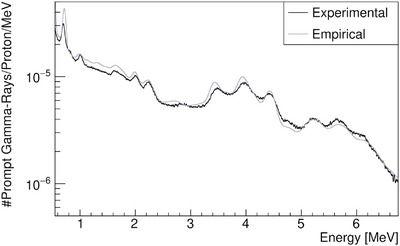
Simulated prompt gamma‐ray spectra in GEANT4 using a model based on experimental and theoretical cross‐sections obtained from the literature.

The results observed in Figures 3, 4, 5 are not satisfactory enough for future research on PGS, especially regarding the oxygen PG line, 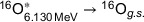. To address this issue, we developed a new model to simulate the de‐excitations resulting from inelastic interactions between protons and carbon and oxygen nuclei. This new model is referred to as the EMPIRICAL model. The new model was integrated into the GEANT4 toolkit due to its open source nature, though it can be implemented in any generic simulator toolkit. The model relies on experimental and theoretical cross‐sections, $\sigma_{PG}$, obtained from the literature,^21^, ^23^, ^24^, ^25^, ^26^, ^27^, ^28^, ^29^, ^30^, ^31^, ^32^, ^33^ particularly when sufficient experimental data is not available. The data from the literature were fitted using a convolution of a *Gaussian* with an exponential decay (see Figures S2 and S3), and the new cross‐section values were used with GEANT4. This model disregards all gamma‐ray emissions resulting from inelastic processes during a G4Step of primary particles and evaluates whether de‐excitation emissions occur. At each step and for each possible gamma‐ray energy of interest, the step length, Δx, and the kinetic energy, $E_{kin}$ of the particle are obtained, then the mean free path, $\lambda =\frac{1}{n\sigma_{PG}\left(E_{kin}\right)}$ (being n, the number density), is determined, followed by the cumulative probability, $1-\exp^{-\frac{\Delta x}{\lambda }}$. Finally, a random number is generated; if the event is accepted, a new secondary gamma‐ray with a specific energy of a de‐excitation line is added to the event for subsequent processing. The resulting PGS spectra for $E_{beam}=110.24\;\mathrm{MeV}$ is depicted in Figure 6.

### 3.4 Quantification of PG spectra lines

**FIGURE 7:**
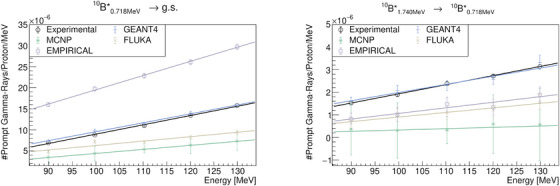
Quantification and comparison of the 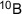 de‐excitation lines observed in the PGS resulting from proton irradiation of PMMA. PAMM, polymethyl‐methacrylate; PGS, Prompt Gamma‐Ray Spectroscopy.

**FIGURE 8:**
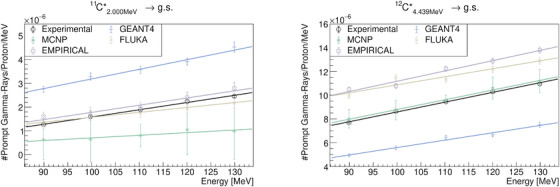
Quantification and comparison of the 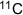 and 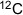 de‐excitation lines observed in the PGS resulting from proton irradiation of PMMA. PAMM, polymethyl‐methacrylate; PGS, Prompt Gamma‐Ray Spectroscopy.

**FIGURE 9:**
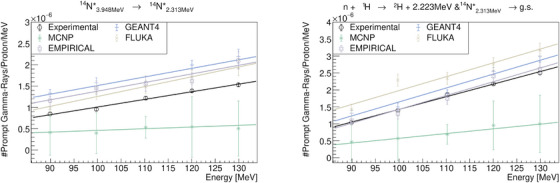
Quantification and comparison of the 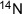 and 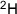 (resulting from neutron capture of 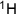) de‐excitation lines observed in the PGS resulting from proton irradiation of PMMA. PAMM, polymethyl‐methacrylate; PGS, Prompt Gamma‐Ray Spectroscopy.

**FIGURE 10:**
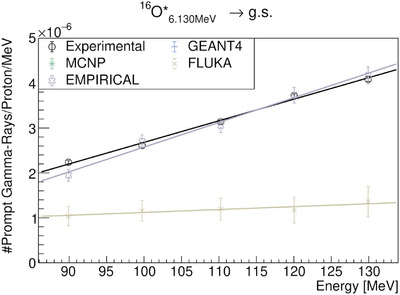
Quantification and comparison of the 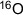 de‐excitation line observed in the PGS resulting from proton irradiation of PMMA. The results of GEANT4 and MCNP6 are suppressed due to their inability to reproduce the 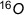 de‐excitation line. PAMM, polymethyl‐methacrylate; PGS, Prompt Gamma‐Ray Spectroscopy.

To quantify the spectral lines in this study, we conducted a baseline restoration using the Sensitive Nonlinear Iterative Peak (SNIP) clipping algorithm.^34^ Subsequently, we selected the region of interest for spectral line analysis and fitted the data with a single or multi‐peak *Gaussian* model. The fitting results were then used to analyze the yield of the lines as a function of beam energy for both experimental and MC data. The most relevant statistical results for the lines are depicted in Figures 7, 8, 9, 10. In general, the peak magnitudes follow the scaling behaviour observed in Figure 2. However, when MC codes fail to accurately reproduce the de‐excitation lines, an uncharacteristic behaviour is observed.

## 4 DISCUSSION

In this study, we conducted a comprehensive analysis of PG spectra resulting from the interaction of protons at energies typically used in clinical settings with a PMMA target. When compared with experimental data, we observed punctual discrepancies in the predicted PG emissions simulated by MCNP6, GEANT4 and FLUKA. To address this discrepancies, a new model, based on experimental and theoretical data from the literature, has been developed for the GEANT4 toolkit.

In the context of PGS analysis, the most prominent PG lines at relatively low energies (less than 1.5MeV) are the 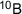 lines. Only the GEANT4 toolkit was able to reproduce these lines (see Figure 7). The discrepancy between the EMPIRICAL model and others are attributed to the lack of experimental data for these energy ranges.^21^ Just, recently experimental data for proton inelastic interactions with carbon have become available in the literature.^31^, ^32^ At energies between 1.5 to 3.0MeV, the 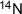 and 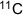 PG lines, and the 2.223MeV gamma‐rays, resulting from neutron capture of 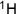, are of particular interest. However, the analyzing these lines is not straightforward due to significant contributions from other reactions. It can be stated that the EMPIRICAL model and FLUKA perform better at these energies, closely following the trends or magnitudes observed in experimental data. At higher energies, the relevant PG lines are the 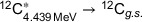 and 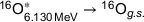 lines. The results for 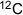, shown in Figure 8, indicate that the gradient of the de‐excitation lines as function of the proton energy is reasonably reproduced by the FLUKA and GEANT4 toolkits, while MCNP6 best reproduces both the gradient and magnitude. Regarding its magnitude, only MCNP6 aligns closely with the experimental data. FLUKA overestimates the line, whereas GEANT4 underestimates it by a factor of approximately 2, consistent with other studies.^35^, ^36^ The EMPIRICAL model does not fully capture the magnitude of the 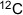 line but shows a good agreement with the experimental gradient. Specifically, when comparing the experimental result of $\left(8.22\pm 0.15\right)\times 10^{-8}\;\mathrm{counts}/\mathrm{MeV}$ to the EMPIRICAL model's results of $\left(8.63\pm 0.71\right)\times 10^{-8}\;\mathrm{counts}/\mathrm{MeV}$, the agreement is evident. This suggests that applying a constant factor could help achieve the desired result. Regarding the oxygen PG line, it is well know that this line is poorly reproduced by MC codes.^3^ The results of the present study are consistent with this observation (see Figure 10). Using the EMPIRICAL model significantly improved the accuracy of the 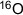 PG line magnitudes, bringing them closer to expected values for the beam energies used in this study.

Across the different MC toolkits, the inelastic cross‐section for proton interactions with 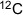 and 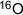 agrees with experimental data.^3^ These processes are based on different physical models leading and influencing the PG emission stage. Furthermore, the PG emission stage is treated in a manner that differs between the various toolkits. In MCNP6, PG emission tends to be more isotropic, closely matching experimental observations. This contributes to its superior performance in the 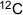 de‐excitation line when compared with the GEANT4 forward direction of PGs.^28^, ^37^ FLUKA and GEANT4, on the other hand, rely on more advanced, adaptable nuclear de‐excitation and photon‐evaporation models.^4^, ^5^, ^6^, ^7^, ^8^ MCNP6 is more library‐dependent and may not offer the same degree of customization as other toolkits. Moreover, the limited energy resolution of the nuclear data tables in MCNP6 limits the the use of data tables in the simulations. In the case of photo‐evaporation process, particularly in the gamma‐ray cascade of 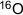, the main transition is an $E_{3}$ transition (through emission of a 6.13MeV photon), as the $E_{0}$ transition (with a energy of 6.05MeV) by single‐photon emission is forbidden for a transition between two states with spin zero, decaying only thought internal conversion.^38^, ^39^ However, in this study and observing the discrete spectra of Figure 4, GEANT4 prominently displays the $E_{0}$ transition in its PG spectra. These discrepancies highlight the need for continuous improvement of the models in these toolkits. In addition, the refining of the model integration of the accurate data tables is essential. For instance while *TENDL* is widely considered as one of the most accurate sources for nuclear reaction data in the low‐energy range, its integration into GEANT4, specifically within the *QGSP_BIC_AllHP* physics list, which is primarily optimized for high‐energy hadronic interactions, may present limitations in certain applications, such as PG production as shown in Figure 4. Although *QGSP_BIC_AllHP* incorporates *TENDL* data for low‐energy reactions, the current integration might not fully capture the complexities of the gamma‐ray production in low‐energy nuclear processes. Therefore, to achieve optimal performance in simulations of low‐energy reactions like gamma‐ray emission, further efforts are needed to refine the model integration and tune the *TENDL* cross‐sections within the framework. While an empirical approach, such as the EMPIRICAL method, can partially mitigate these discrepancies, further advancements in the physical models would enhance the predictive power and precision of the 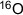 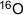 PG emission MC simulations in proton therapy.

## 5 CONCLUSION

Given the crucial role of MC simulations in the progress of range verification techniques, our study focused on using MCNP6, GEANT4 and FLUKA to simulate PGs. We chose this approach because MC simulations are fundamental to the development and effective application of these techniques. Our investigation revealed specific discrepancies in MC results for relevant de‐excitation lines, particularly those associated with carbon, and oxygen, across different toolkits. Additionally, we applied a new model based on experimental and theoretical cross sections, which improved the reproduction of the oxygen de‐excitation line that is not correctly observed by any of the MC toolkits. The findings of this study highlight the need to update the data tables of MC simulations, and imply a clear need for further theoretical and experimental investigations of PGs de‐excitation lines resulting from nuclear reactions relevant to proton therapy. The models developed here can be used in any study to investigate secondary gamma‐rays when PMMA is used as a tissue‐equivalent material.

## CONFLICT OF INTEREST STATEMENT

The authors declare no conflicts of interest.

## Supporting information

Supporting Information
