# Supplementary material for: A comparative analysis of GEANT4 , MCNP6 and FLUKA on proton‐induced gamma‐ray simulation
Source: Med Phys. 2025 Mar 11;52(6):4862–70. doi: 10.1002/mp.17754 (PMC12149693; doi:10.1002/mp.17754)
Supplement: Supplementary file 1 — Supporting Information [file MP-52-4862-s001.pdf]

VII. Supporting Material

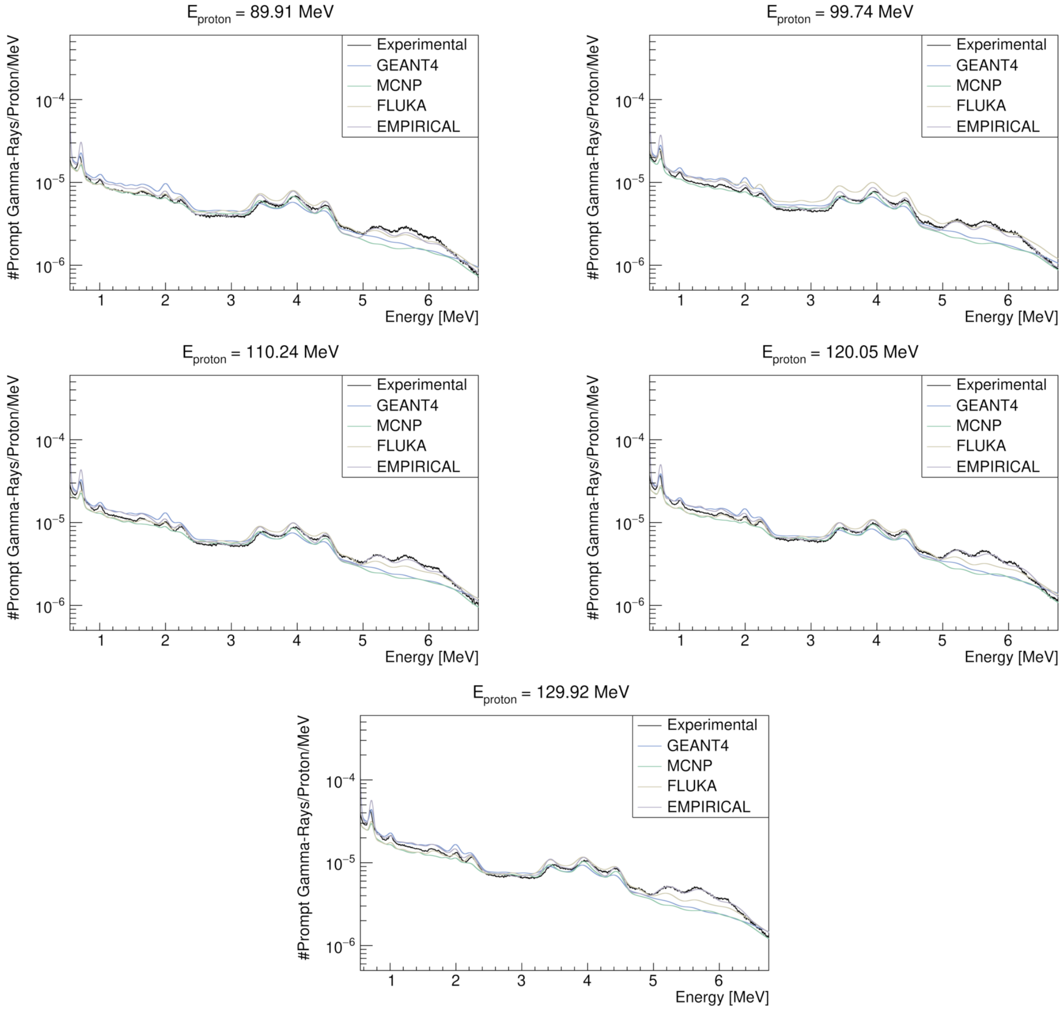

Figure S-1: Prompt gamma-ray spectra resulting from proton irradiation of PMMA (Experimental) compared to simulated prompt gamma-ray spectra in `MCNP6` (*ISABEL*), `GEANT4` (*QGSP_BIC_HP*) and `FLUKA` (*PRECISION*) for the models or physics lists that best fit the experimental data. Results for all proton energies (E_{proton}) used in this study.

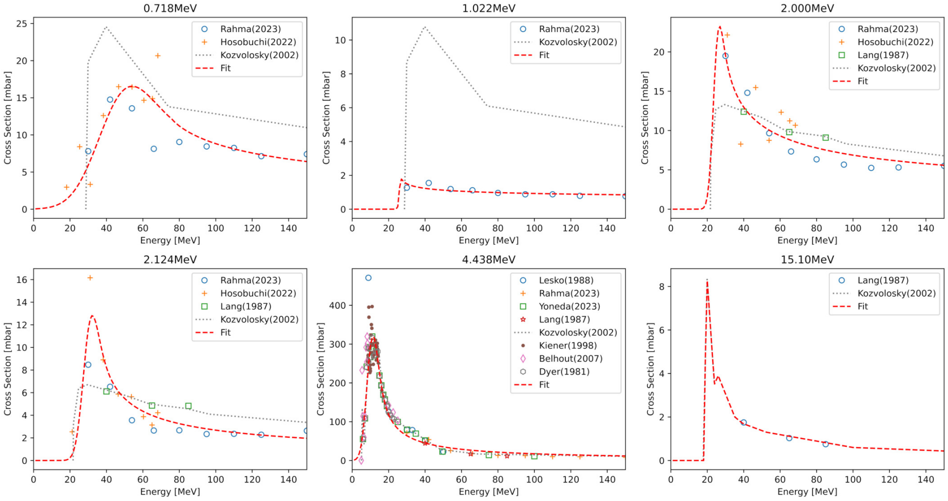

Figure S-2: The analysis of carbon proton inelastic cross-sections de-excitation lines was used to derive the results corresponding to the *EMPIRICAL* data. The red dashed line represents the fitted values (based on literature results[21,23,24,25,26,27,28,29,30,31,32,33]) used in `GEANT4`.

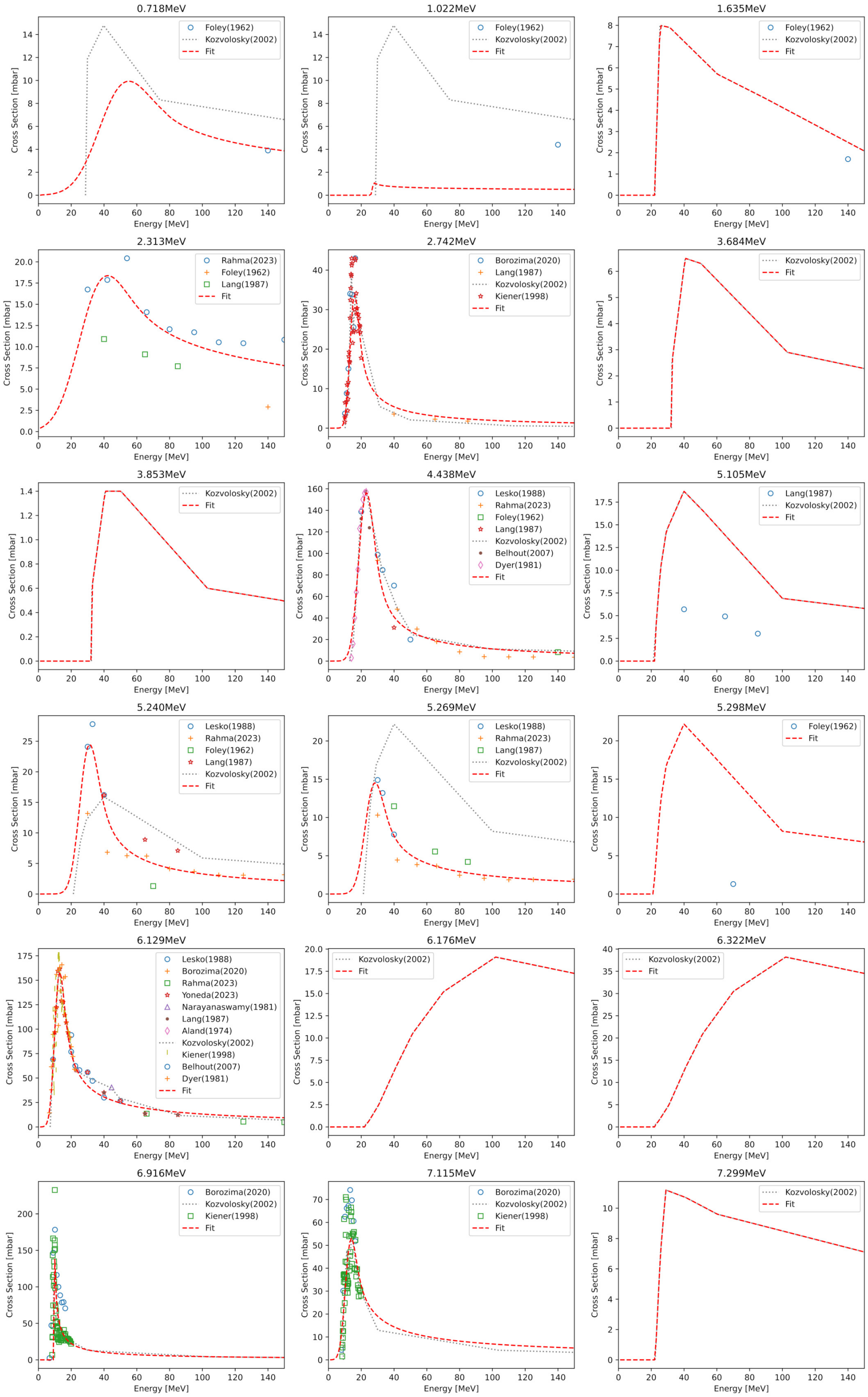

Figure S-3: The analysis of oxygen proton inelastic cross-sections de-excitation lines was used to derive the results corresponding to the *EMPIRICAL* data. The red dashed line represents the fitted values (based on literature results[21,23,24,25,26,27,28,29,30,31,32,33]) used in `GEANT4`.
